# How Climatic Seasons of the Amazon Biome Affect the Aromatic and Bioactive Profiles of Fermented and Dried Cocoa Beans?

**DOI:** 10.3390/molecules26133759

**Published:** 2021-06-22

**Authors:** Daniela Pinheiro Gaspar, Gilson Celso Albuquerque Chagas Junior, Eloisa Helena de Aguiar Andrade, Lidiane Diniz do Nascimento, Renan Campos Chisté, Nelson Rosa Ferreira, Luiza Helena da Silva Martins, Alessandra Santos Lopes

**Affiliations:** 1 Laboratório de Processos Biotecnológicos (LABIOTEC), Programa de Pós-graduação em Ciência e Tecnologia de Alimentos (PPGCTA), Instituto de Tecnologia (ITEC), Universidade Federal do Pará (UFPA), Campus Guamá, Belem 66075-110, Para, Brazil; gilsonjr.bra@gmail.com (G.C.A.C.J.); rcchiste@ufpa.br (R.C.C.); nelson.ufpa@gmail.com (N.R.F.); 2 Centro Universitário do Estado do Pará (CESUPA), Centro de Ciências Biológicas e da Saúde, Curso de Nutrição, Campus Nazaré, Belem 66035-135, Pará, Brazil; 3 Laboratório Adolpho Ducke, Coordenação de Botânica, Museu Paraense Emílio Goeldi, Av. Perimetral, 1900, Terra Firme, Belem 66077-830, Para, Brazil; eloisa@museu-goeldi.br (E.H.d.A.A.); lidianenascimento@museu-goeldi.br (L.D.d.N.); 4 Faculdade de Engenharia de Alimentos (FEA), Instituto de Tecnologia (ITEC), Universidade Federal do Pará (UFPA), Campus Guamá, Belem 66075-110, Para, Brazil; 5 Instituto da Saúde e Produção Animal (ISPA), Universidade Federal Rural da Amazônia (UFRA), Av. Perimetral, 2501, Campus Belém, Terra Firme, Belem 66077-830, Para, Brazil; luiza.martins@ufra.edu.br

**Keywords:** GC-MS, *Theobroma cacao*, epicatechin, identity standard, theobromine, chocolate

## Abstract

In addition to the vast diversity of fauna and flora, the Brazilian Amazon has different climatic periods characterized by periods with greater and lesser rainfall. The main objective of this research was to verify the influence of climatic seasons in the Brazilian Amazon (northeast of Pará state) concerning the aromatic and bioactive profiles of fermented and dried cocoa seeds. About 200 kg of seeds was fermented using specific protocols of local producers. Physicochemical analyzes (total titratable acidity, pH, total phenolic compounds, quantification of monomeric phenolics and methylxanthines) and volatile compounds by GC-MS were carried out. We observed that: in the summer, the highest levels of aldehydes were identified, such as benzaldehyde (6.34%) and phenylacetaldehyde (36.73%), related to the fermented cocoa and honey aromas, respectively; and a total of 27.89% of this same class was identified during winter. There were significant differences (*p* ≤ 0.05, Tukey test) in the profile of bioactive compounds (catechin, epicatechin, caffeine, and theobromine), being higher in fermented almonds in winter. This study indicates that the climatic seasons in the Amazon affect the aromatic and bioactive profiles and could produce a new identity standard (summer and winter Amazon) for the cocoa almonds and their products.

## 1. Introduction

The chocolate manufacturing stages are well defined; however, depending on the variety of cocoa, the techniques employed and the climatic conditions, the fermented seed can be influenced by the profiles of flavor, acidity, and antioxidant capacity [1].

The fermentation of cocoa beans occurs on the producing farms, where the aromatic precursor characteristics of chocolate are formed. There is also a reduction in acidity, phenolic compounds, and methylxanthines responsible for the natural bitterness and astringency of the seeds. Fermentation occurs spontaneously, for 5 to 7 days. In Brazil, seeds are fermented in wooden boxes and upturned every 24 h to favor the microbial dynamics and drainage of liquids formed in the first days of fermentation [2,3,4].

During the fermentation of cocoa, several biochemical transformations essential for the formation of precursors of chocolate aromas occur [5,6,7]. In this sense, aldehydes, esters, ketones, and alcohols are the results of the metabolism of certain yeast species that act spontaneously in the first days of fermentation, such as those of the genus *Saccharomyces*, *Pichia*, and others [8,9,10,11]. Once formed, some compounds are degraded, volatilized, or participate in chemical and biochemical reactions in later stages, such as drying and roasting [9,12]. These aromatic compounds are responsible for imparting fruity, sweet, and floral notes typical of good quality chocolates, thus being well-received in the market. Several important sensory molecules come from the metabolism of the microbial population that participates in the fermentation process, mainly due to the participation of yeast species [5,9].

The first study on volatile compounds in fermented cocoa seeds in the Brazilian Amazon [9] reported a considerable diversity of aromatic compounds from different classes (alcohols, aldehydes, ketones, and ethers) after the fermentation process started with applying starter cultures of *S. cerevisiae* and *P. kudriavzevii:* 2-Nonanol, Phenylacetaldehyde, Benzaldehyde, 2-Nonanone, 2-Phenethyl acetate, and Isoamyl benzoate. All of them are the result of the metabolism of the microbial population that includes several species and yeasts, and lactic and acetic bacteria [5,9].

The seeds naturally contain a considerable amount of astringent, bitter, and bioactive compounds, such as catechin, epicatechin, theobromine, and caffeine, even after fermentation. Bioactive compounds are degraded during fermentation through the action of enzymes, high temperature, and upturning. However, high levels of these compounds can indicate poorly conducted fermentations. Pharmacologically it would be interesting to have these compounds at levels suitable for consumption without impairing sensory quality [13,14].

The region where the experiment was carried out (state of Pará) corresponds to the high cocoa production in Brazil (135,000 t. in 2019) [15]. In this region, there is a high annual rainfall variation characterized by two periods popularly known as Amazonian summer and winter [16].

Thus, considering that the region’s climate is an edaphoclimatic factor that can influence the cocoa fermentation process, the objective of this study was, for the first time, to characterize spontaneously fermented almonds during the two climatic periods in the Brazilian Amazon. For this, the physicochemical parameters (pH, acidity, phenolic compounds, and methylxanthines) and aromatic profile were evaluated. There is no knowledge about the climatic influence on cocoa fermentation in the Brazilian Amazon, so this research also aims to enrich the specialized literature on the subject and propose improvements in the control of the fermentation process in the region in other locations.

## 2. Results

### 2.1. Physicochemical Analysis in Fermented and Dried Cocoa Beans

The pH values of fermented and dried cocoa beans from summer (4.09) and winter (4.07) were statistically similar (*p* ≥ 0.05). Therefore, the season of the year in which the cocoa is fermented and dried did not influence this parameter (Table 1).

On the other hand, statistically different results were obtained (*p* ≤ 0.05) when referring to the total acidity of cocoa beans. The values found in summer (18.72 mEq NaOH/100 g) and in winter (20.01 mEq NaOH/100 g) were higher than the range indicated by the Cocoa Index (10.53–19.37 mEq NaOH 100/g) to obtain superior-quality almonds [17]. Total acidity, which measures the total concentration of acids in the food, is a great predictor of flavor [18].

In this study, the levels of total phenolic compounds in fermented and dried almonds differed statistically from each other (*p* ≤ 0.05); in winter there was a value of 25.20 mg ECE/g and in summer the value was 30.89 mg ECE/g (Table 1). Therefore, it seems the season of the year has a relevant influence on the content of total phenolic compounds.

The values of theobromine in summer (8.50 mg/g) and winter (8.37 mg/g), and caffeine in summer (2.55 mg/g) and winter (2.45 mg/g), are statistically equal, indicating that has no influence of the season of the year when cocoa is grown and fermented, on these parameters (Table 1).

Statistically analyzing the results of catechin and epicatechin in the two periods, it is observed that there was a significant difference (*p* ≤ 0.05) for the dried fermented almonds, making it possible then to infer these compounds vary according to the season.

### 2.2. Profile of Volatile Compounds in Fermented and Dried Cocoa Beans

In this research, 21 compounds in fermented and dried cocoa beans were identified in the 2 climatic different periods grouped into 4 classes. The esters were in a higher amount in the number of constituents (8), followed by ketones (3) aldehydes (2), and alcohols (2) (Table 2).

**Figure 1:**
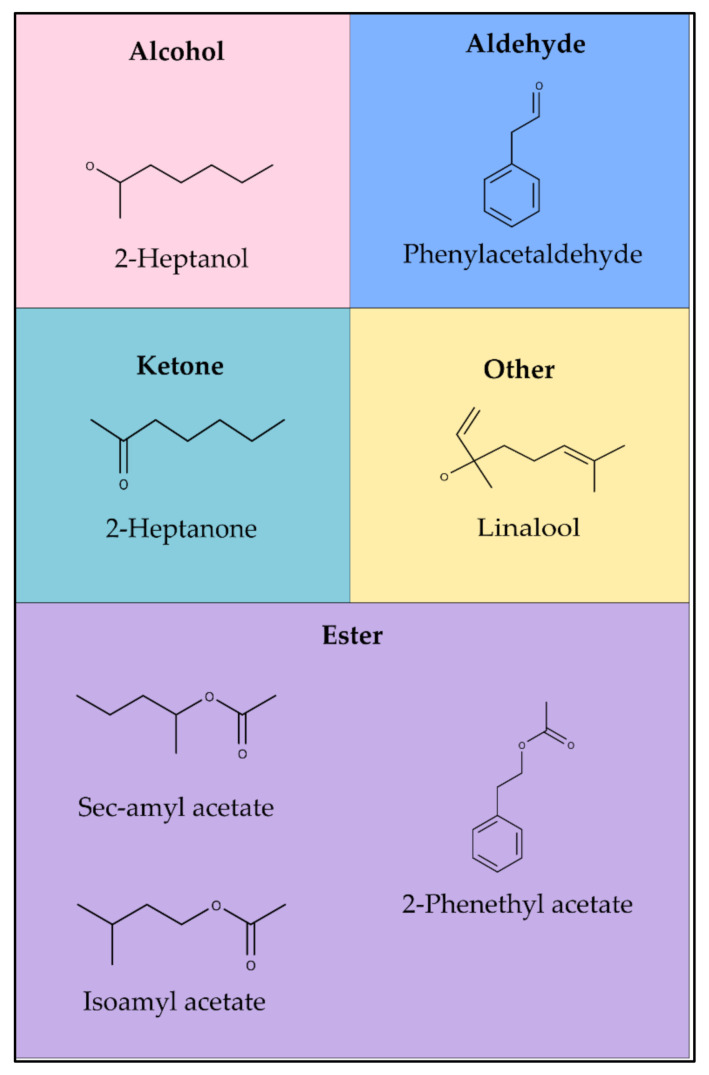
Major volatile compounds identified in fermented and dried cocoa beans in Inhangapi, PA, Brazil (2017–2018).

The major compounds found in fermented and dried cocoa in the summer season were the aldehyde phenylacetaldehyde and the esters isoamyl acetate and 2-phenethyl acetate. The sensory characteristics of those constituents are associated with honey, fruit, and roses. During the winter season, the fermented and dried cocoa presented floral, honey, and banana characteristics derived from the linalool, phenylacetaldehyde, and sec-amyl acetate compounds alcohol, aldehyde, and ester, respectively (Figure 1).

In this study, no pyrazines compounds were identified, which are highly desirable (in ideal quantities) for promoting the characteristic chocolate flavor [9]. However, this finding can mean a positive differential in the aromatic quality of the chocolate produced with these almonds since several compounds with fruity, floral, and sweet attributes have been identified.

## 3. Discussion

The pH values are also an important parameter for the cocoa quality. The Cocoa Index establishes that pH values between 5.60 and 6.57 are considered almonds with good quality for cocoa beans [17].

The low number of polyphenols found in cocoa at the end of fermentation and drying is due to the complex enzymatic reactions that occur from the breakdown of cell membranes, causing the oxidation of polyphenols by polyphenoloxidase enzyme (PPO). This reaction changes the color of cocoa seeds from violet to brown [25]. The coloring of fermented and dried cocoa beans is the main quality parameter used by the chocolate industries, which seek well-fermented cocoa (brown color), and reject violet-colored cocoa. They indicate that fermentation was not appropriately conducted, resulting in almonds with a bitter taste [26].

Even if the contents of polyphenols drastically decrease in the almond, that will benefit the industry, and it reduces even more when cocoa by-products are made (chocolate bars, cocoa powder, and other derivatives); these foods are seen as a good source of phenolic antioxidants for some populations. It is possible to control the chocolate production process and preserve up to 70% of the phenolic compounds [27,28]. In addition, some authors [29] proved that cocoa has more phenolic compounds and a greater antioxidant capacity than products recognized for these properties, such as teas and red wine.

The increase in solar incidence is proportional to the increase in the levels of secondary metabolites for plant protection, such as phenolic compounds [30]. Research [31] highlighted other factors, such as the age of the cocoa tree and the chemical compositions of the soil, which can influence the concentration of secondary metabolites.

This divergent behavior to that found in the literature [32,33] can also be explained by the decrease in the synthesis of PPO in cocoa beans in winter, compared to summer. Thus, in the final fermentation stage, where phenolic compounds are partially oxidized by PPO, cocoa beans fermented in winter would be less influenced by the enzymatic degradation of phenolic compounds than in summer. These enzymes can exhibit different behaviors and stability during fermentation and can be inactivated by several factors, such as heat and acidity [34]. In addition, the reactions between compounds from different compartments, such as what happens between PPO and polyphenols, can be prevented by the fusion of lipid vacuoles, which in this way makes impossible the contact between enzyme and substrate [35].

Data from this study are in agreement with Matissek [36], who reported that theobromine and caffeine are responsible for approximately 99% of methylxanthines and that theobromine is the main methylxanthine in cocoa. In contrast, caffeine is present in small amounts and theophylline in insufficient quantities or not found in cocoa beans. Theobromine is present in higher concentrations in cocoa because it is one of the precursors of the caffeine pathway (through a methylation process), and this conversion of theobromine to caffeine is a slow process. The reason theophylline does not appear is still due to the conversion of caffeine into theophylline through a reaction catalyzed by a demethylase [37].

In this study, theobromine and caffeine contents were similar to those found by Chagas Junior et al. [14]. In comparison with the study by Carrillo et al. [33], only the theobromine contents were similar, as the caffeine content of this study was higher than that found, a fact that can be explained by the difference in the type of cocoa used in fermentation and the fermentation method established on the farm.

Regarding the volatile compounds, aldehydes are found quite often in food [38] and have a shallow aroma threshold. The C3–C5 aldehydes, such as propanal, butanal and pentanal, have a malted/green characteristic, with notes of malted and bitter cocoa used in malt and chocolate aromas. In this study, the major aldehyde in the fermented and dried cocoa was phenylacetaldehyde, present at high concentrations in both periods. Phenylacetaldehyde is a derivative of the amino acid catabolism during cocoa fermentation [19].

Esters are formed from alcohols and acids, and play a significant role forming the taste of foods, and their aroma will depend on their molecular structure and conformation. Those with low molecular weight can be easily found in fruits, flowers, and fermented drinks, and exhibit a characteristic fruity aroma. However, as the chain increases, the odor may become more sweet, metallic, or similar to soap [39]. The esters found in the spontaneous cocoa fermentation in this study were more observed in summer than in winter, and 2-Phenethyl acetate and isoamyl acetate present odors of roses and balsam, respectively.

Alcohols are compounds that play an essential role in the flavor formation of food and are widely distributed in nature, and almost all have desirable and pleasant aromas. Its hydroxyl group determines most of its properties, completely changing the odor when modifications occur in this group. Straight-chain alcohols are abundant in fruits, often increasing with maturity [40]. Amyl alcohols are found in the cocoa fermentation process and some of them are used to evaluate the taste and fermentation of cocoa [41]. The main alcohols found were 2-heptanol and 2- Phenylethyl ethanol, with sweet and floral flavor characteristics in the present research.

Ketones formed through oxidation and decarboxylation of fatty acids are well known as food odorants due to their aroma similar to nuts [39]. The 2-heptanone compound, which has floral and fruity characteristics, was the major compound of this group, and it was present in higher levels in the winter season.

These classes are directly related to the metabolism of the various yeast species that are naturally found during the first days of cocoa fermentation [5], but the aromatic profile can be differentiated because some compounds are specific to certain yeasts [9].

Some authors [42] reported the correlation of linalool and 2-nonanone with fine cocoa, as these compounds were found in Criollo cocoa, which is used to make cocoa by-products of more excellent commercial value. Linalool and 2-nonanone were found at higher concentrations in winter than in summer, with the concentration of linalool being about 8 times higher, while 2-nonanone shows that their concentration was 10 times higher than the concentration found in summer. Thus, cocoa produced in winter has a more significant potential to produce fine cocoa than summer cocoa, and the seasons might interfere in the marketing potential of cocoa beans in the Amazonian region.

It is important to control the cocoa fermentation time to avoid the formation of undesirable volatile compounds (off-flavors) that can be found if the fermentation exceeds the necessary time, as the excess fermentation can lead to a significant increase in bacilli and filamentous fungi that produce off-flavors [7].

Rancid, waxy or sulfurous aromas are potentially harmful to the aroma of cocoa and come from propanoic acid, cyclohexanol, and thiobis-methane, which were reported by some authors [42] as a result of the excess fermentation of Criollo cocoa. The National variety produced the substances thiobis-methane and naphthalene reported as sulfur and pungent aroma. Forastero cocoa produced butanoic acid, hexanoic acid, pyridine, heptanoic acid, and peracetic acid, which are undesirable because they have fat and a rancid aroma. In this study, these off-flavor compounds were not detected.

The taste of cocoa and its by-products is strongly influenced by the cocoa genotype and the chemical and microbial reactions that occur during fermentation and industrial processing. The precursors of flavor and aroma are formed during the fermentation and drying of the cocoa beans, and roasting, subsequent thermal treatment in industries, is also essential for the formation of quality cocoa [43]. The volatile compounds of cocoa beans are considered one of the most important indicators for assessing the quality of cocoa, and they are also influenced by factors such as climatic conditions of cocoa growth and soil composition [31].

Recent studies indicate that cocoa fruits are directly influenced by the climate in the formation of aromatic compounds, showing that this formation is favored during rainy seasons due to the intensity of the rains and a higher frequency of clouds, which can increase the energy of the plant for the synthesis of characteristic aromas of cocoa. On the other hand, there may be more water withdrawal from the plant during the season with less rainfall, which affects the aromatic profile [44].

In this study, behavior contrary to that reported [44] is observed, which shows more significant amounts of aromatic compounds during the drier season. This can be associated with the fact that there is a considerable amount of precipitation in the drier season in the Brazilian Amazon region, suggesting that there is a balance in the time of exposure to sunlight and rainfall in the region.

In this study, microbiological analyses were not carried out during the fermentation processes, but the importance of knowing the existing microbial dynamics and identifying the population of yeasts and bacteria naturally existing in the process is emphasized. They are responsible for initiating aromatic compounds and triggering essential reactions inside the cocoa seed to promote changes in acidity, the content of bioactive compounds, and temperature.

This research is necessary because it opens the door for future more comprehensive studies on the subject and proposes measures to understand the solar and rainfall effects in the region, as it is the site of the highest cocoa productivity in Brazil, positively influencing and highlighting the region and the country in the international cocoa market.

## 4. Materials and Methods

### 4.1. Chemicals and Reagents

For the physicochemical analysis, reagents with analytical grade were used: petroleum ether (≤100%, Dinâmica, Indaiatuba, SP, Brazil), Folin–Ciocalteau reagent (Êxodo Científica, Sumaré, SP, Brazil). Pentane (≥99%) for the GC-MS analysis and the standards of (+)-catechin (≥99%), (−)-epicatechin (≥98%), theobromine (≥98%), theophylline (≥99%) and caffeine (≥99%) for the spectrophotometric and HPLC methods, were purchased from Sigma-Aldrich Chemical Co. (St. Louis, MO, USA).

For the HPLC analysis, we used methanol HPLC grade (≥99%, J.T. Backer, Avantor Materials, PA, USA), acetonitrile HPLC grade (Dinâmina) and the ultrapure water was obtained from Milli-Q system (Millipore Corp, Milford, MA, USA). All the solvents were filtered through 0.45 µm nylon membrane (FilterPro), and the extracts for the injection were filtered with 0.22 µm syringe filters (Analítica, São Paulo, SP, Brazil).

### 4.2. Material

Cocoa fruits, variety Forastero, were harvested in Inhangapi city, PA, Brazil (1°25′48″S, 47°55′1″W) during October 2017 and February 2018. Traditionally in this region, these months are characterized by the occurrence of less and more frequent rainfall, respectively. This characteristic receives the popular denomination of *Amazonian summer* and *Amazonian winter*, respectively.

### 4.3. Fermentation of Cocoa Beans and Artificial Drying

The fermentation process took place on a cocoa-producing farm with its own protocols and was not influenced by the researchers.

The cocoa fruits were harvested and remained at rest for 48 h under direct sunlight and rain, as some studies explain that a period between the harvest and the opening of the cocoa fruit can prevent some unwanted reactions such as the fruit over-ripening [45]. After this period, the fruits were opened manually, with the pulp seeds separated from the placenta and the peels.

The seeds (approximately 200 kg) were transferred to wooden boxes (0.43 m^3^), with the practice of turning the seeds (at intervals of 24 h, after the first 48 h) until the completion of the process, which lasted 6 days. The seeds were covered with banana leaves and burlap sacks to maintain the obtained temperature. The process was carried out in duplicate.

Small portions of seed samples (400 g) were removed for physicochemical analysis and stored at −18 °C. The artificial drying process was carried out to simulate the natural drying carried out on the farm to reduce moisture to values of 6%, which is recommended to avoid the proliferation of molds and the obtaining of very brittle almonds [46].

### 4.4. Methods

#### 4.4.1. Physicochemical Analysis of Cocoa Beans

The fermented and dried cocoa beans were ground in an analytical mill (model A11B, Ika, Satufen, Germany) for the determination of total titratable acidity (TTA, method 31.06.06) and pH (method 970.21), according to the Association of Official Analytical Chemists [47]. All analyses were performed in triplicate.

#### 4.4.2. Determination of Total Phenolic Compounds in Fermented and Dried Cocoa Beans

Samples of fermented and dried cocoa beans were lyophilized (L101 Liotop, São Paulo, SP, Brazil) and defatted with petroleum ether [48]. Then, the total phenolic compounds were determined by the Folin–Ciocalteau colorimetric assay [49] in an UV-vis spectrophotometer (model EVO 60, Thermo Fisher Scientific, Waltham, MA, USA) at 760 nm and the readings interpolated in curve of epicatechin standard (Range concentration 20–100 mg/L, R^2^ ≥ 0.99). The results, in duplicate, were expressed in milligram equivalent epicatechin per gram sample (mg ECE/g).

#### 4.4.3. Determination of Methylxanthines and Monomeric Compounds by HPLC-DAD

Defatted samples (250 mg) of fermented and dried beans were subjected to extraction of methylxanthines (theobromine, theophylline and caffeine) and monomeric phenolic compounds (catechin and epicatechin), according to previous studies [14,50,51]. Aliquots of the extracts (20 µL) obtained were injected into a chromatograph equipped with a diode array detector (HPLC-DAD) at 280 nm (1260 Infinity Agilent Technologies, La Jolla, CA, USA) with Zorbax Eclipse XBD-C18 column (4.6 × 150 mm, 5 µm, Agilent Technologies, La Jolla, CA, USA) at 25 °C, at flow rate of 1.2 mL/min of mobile phases (A) water/acetonitrile (99.8: 0.2, *v*/*v*) and (B) methanol, in gradient mode according Chagas Junior et al. [14].

The tentative identification of the compounds was based on the retention time of each analyzed standard and quantified through the external analytical curves: theobromine (from 3.125to 50 µg/mL, R^2^ ≥ 0.99, LOQ = 0.64 mg/g), theophylline (from 3.125to 100 µg/mL, R^2^ ≥ 0.99, LOQ = 0.02 mg/g), caffeine (from 3.125 to 100 µg/mL, R^2^ ≥ 0.99, LOQ = 0.04 mg/g), catechin (from 3.125 to 50 µg/mL, R^2^ ≥ 0.99, LOQ = 0.31 mg/g) and epicatechin (from 3.125to 100 µg/mL, R^2^ ≥ 0.99, LOQ = 0.11 mg/g) [11]. All experiments were performed in duplicate.

#### 4.4.4. Extraction and Identification of Volatile Compounds in Fermented and Dried Cocoa Beans by GC-MS

The fermented and dried cocoa beans were milled (A11B, Ika) and subjected to simultaneous distillation/extraction for 2 hours using 4 mL of pentane as a solvent. The volatile concentrate obtained was stored at a temperature of 5 °C [52,53,54].

Aliquots (2 μL) of volatile concentrate was injected into a gas chromatograph coupled to mass spectrometry (GC-MS), in a Shimadzu QP-2010 Plus system, equipped with a DB-5MS column (30 m × 0.25 mm × 0.25 μm). The helium gas was used as the carrier gas with a flow rate of 1.2 mL/min. The temperature of the injector and the interface was 250 °C, and the oven temperature was adjusted to 60–250 °C, using a ramp of 3 °C/min. Electronic impact mass spectrometer at 70 eV and the ion source temperature at 220 °C were used.

Quantitative analysis of the chemical constituents was performed by peak-area normalization using a flame ionization detector (FID—Shimadzu, QP 2010 system) under the same conditions as GC-MS, except that hydrogen was used as a mobile phase. Chemical identification was carried out by comparing the mass spectra and retention indices (RI) with those of standard substances in the system libraries and with data from the literature [55,56,57]. The RIs were obtained using a homologous series of *n*-alkanes (C8–C24, Sigma-Aldrich Co, St. Louis, MO, USA).

### 4.5. Statistical Analysis

The results of the physicochemical analyzes were submitted to Analysis of Variance (ANOVA) and compared by Tukey test (*p* ≥ 0.05) performed with the software Statistica 7.0 (StatSoft Inc., Tulsa, OK, USA).

## 5. Conclusions

The two climatic periods present in the Amazon region were responsible for distinguishing the physicochemical characteristics and the aromatic profile of fermented and dried cocoa beans. In the period with less rainfall (*Amazonian summer*), less acidic almonds with lower levels of phenolic compounds were obtained, which may favor the reduction of acidity, bitterness, and astringency for chocolate.

Regarding the aromatic profile, in the summer, there was a greater concentration and variety of desirable compounds to the cocoa almond, which can confer floral, fruity, and sweet notes to the chocolate, thus emphasizing the possibility of obtaining chocolate with differentiated notes in the Amazon region.

Future studies of molecular biology and sensory analysis in the chocolate obtained can answer the biological questions of the formation of certain aromatic compounds and differentiate them about specific attributes of the product.

## Figures and Tables

**Table 1:** Physicochemical analyses in fermented and dried cocoa beans during the Amazonian summer and winter (Inhangapi, Pará, Brazil, 2017–2018).

	Treatments/Means *	
Parameters	Summer	Winter
pH	4.09 ± 0.01 ^a^	4.07 ± 0.01 ^a^
TTA (meq. NaOH/100 g) ^1^	18.72 ± 0.11 ^b^	20.01 ± 0.38 ^a^
Total Phenolic Compounds (mg ECE/g) ^2^	25.10 ± 0.12 ^b^	30.89 ± 0.75^a^
**Monomeric Compounds**		
Catechin (mg/g)	0.37 ± 0.00 ^b^	0.41 ± 0.01 ^a^
Epicatechin (mg/g)	0.53 ± 0.01 ^b^	1.12 ± 0.01 ^a^
**Methylxanthines**		
Theobromine (mg/g)	8.50 ± 0.18 ^a^	8.37 ± 1.54 ^a^
Caffeine (mg/g)	2.55 ± 0.05 ^a^	2.45 ± 0.25 ^a^

**^1^** meq. NaOH/100 g: milliequivalent sodium hydroxide per 100 g per sample. **^2^** mg ECE/g: milligram equivalent epicatechin per gram sample. * Means ± standard deviation with different letters in the same line (climatic season) are statistically different (Tukey test, *p* ≤ 0.05).

**Table 2:** Profile of volatile compounds in fermented and dried cocoa beans during the Amazonian summer and winter (Inhangapi, Pará, Brazil, 2017–2018).

Group	RI *	Compound	Summer Area %	Winter Area %	Attribute **
Alcohols	894	2-Heptanol	3.52	6.78	Sweet, citrus
	1106	2-Phenylethyl ethanol	0.35	0.30	Honey, floral, caramel
		**Total Alcohols (%)**	**3.87**	**7.08**	
Aldehydes	952	Benzaldehyde	6.34	2.44	Bitter almonds, grass
	1036	Phenylacetaldehyde	36.73	25.45	Honey
		**Total Aldehydes (%)**	**43.07**	**27.89**	
Ketones	889	2-Heptanone	3.88	5.14	Floral, fruity
	1059	Acetophenone	2.29	2.18	Floral, almonds, sweet
	1087	2-Nonanone	2.40	4.16	Floral
		**Total Ketones (%)**	**8.57**	**11.48**	
Esters	869	Isoamyl acetate	9.41	4.64	Fruity
	871	Sec-amyl acetate	5.89	9.63	Banana
	1169	Ethyl benzoate	0.46	0.21	Floral, fruity, chamomile
	1196	Ethyl octanoate	0.40	0.30	Floral, fruity
	1243	Ethyl phenylacetate	1.50	0.81	Sweet, waxy
	1251	2-Phenethyl acetate	15.38	4.68	Roses
	1433	Isoamyl benzoate	3.03	4.15	Balsam, sweet
	1594	Ethyl dodecanoate	0.06	0.23	Floral, fruity
		**Total Esters (%)**	**36.13**	**24.65**	
Others	988	Mycrene	0.93	6.75	Off-flavor
	1032	Cis-beta-ocimene	0.37	3.30	Floral
	1095	Linalool	3.99	11.14	Floral
	1128	Allo-ocimeno	0.23	2.47	Floral
	1200	*n*-Dodecane	0.22	0.24	Off-flavor
	1400	*n*-Tetradecane	0.26	0.39	Off-flavor
		**Total Others (%)**	**6.00**	**24.29**	
		**Total (all groups) (%)**	**97.64**	**95.39**	

*: Retention index calculated from a series of *n*-alkanes (C8–C40) in column DB-5MS. **: Characteristics found in the literature [5,19,20,21,22,23,24].

## Data Availability

Data is contained within the article.

## Sample Availability

Samples of the compounds of the fermented and dried cocoa beans are available from the authors.

## Abbreviations

ANOVA	Analysis of Variance
FID	Flame Ionization Detector
GC-MS	Gas chromatograph coupled to mass spectrometry
LOQ	Limit of quantification
mEq NaOH/100 g	milliequivalent sodium hydroxide per 100 g sample
mg ECE/g	milligram equivalent epicatechin per gram sample
PPO	Polyphenoloxidase
RI	Retention index
